# Topo-phylogeny: Visualizing evolutionary relationships on a topographic landscape

**DOI:** 10.1371/journal.pone.0175895

**Published:** 2017-05-01

**Authors:** Jamie Waese, Nicholas J. Provart, David S. Guttman

**Affiliations:** 1 Department of Cell & Systems Biology, University of Toronto, Toronto, Ontario; 2 Centre for the Analysis of Genome Evolution & Function, University of Toronto, Toronto, Ontario; University of Colorado, UNITED STATES

## Abstract

Phylogenetic trees are the de facto standard for visualizing evolutionary relationships, but large trees can be difficult to interpret because they require a high cognitive load to identify relationships between multiple operational taxonomic units (OTUs). We present a new tool for displaying phylogenetic relationships as a topographic map in which OTUs autonomously attract or repel one another based on their individual branch lengths and distance to a common ancestor. This data visualization paradigm makes it possible to preattentively identify the nature of the relationship between items without having to trace a complex network of branches back to the root. This tool was developed for exploring phylogenetic data, but the technique could be extended for visualizing other hierarchical structures as well.

## 1 Introduction

Phylogenetic trees and cluster diagrams are essential tools for displaying evolutionary and similarity relationships among species, gene or protein sequences, and many other types of data. These tree-like branching diagrams are generally intuitive and easy to read, but they do not scale well when working with very large numbers of operational taxonomic units (OTUs). Problems arise due to a poor overall use of space, arbitrary clade ordering, hard to read labels (especially with radial trees), and the high cognitive load required for tracing lineages to their common ancestral nodes to determine lineage and clade relationships. While these are trivial issues when working with most moderately size trees, they can become problematic when the trees become very large, or with trees that show early diversification and long terminal branches. Despite these problems, phylogenetic trees remain the de facto standard for displaying evolutionary relationships, despite very little work exploring the effects of this visualization approach on understanding [1].

Several tools have attempted to address these issues, for example PhyloMap [2], TreeJuxtaposer [3], TreeWiz [4], Dendroscope [5], Treevolution [6], PAUP [7], Mesquite [8], ToLWeb [9], FigTree [10], PhyloWidget [11], OneZoom [12] and others. An impressive list of 392 different phylogeny visualization and analysis packages is maintained by Felsenstein [13]. These tools enable users to zoom in and out of branches, compress clades into groups and produce circular trees and other layouts that display a large amount of information in a limited space. Hughes et al [14] developed a unique method for displaying large phylogenetic trees in 3D hyperbolic space, taking advantage of an additional dimension to visualize and navigate more than 100,000 nodes. However each of these approaches maintains the underlying tree metaphor that requires the viewer to use “top down” cognitive visual processes [15] to determine the nature of a relationship between two OTUs. Simply put, trees require conscious direction of the gaze as it travels up and down the network to determine how two OTUs are linked.

Trees are not the only way to visualize phylogenetic relationships. Isabel Meirelles [16] categorizes visual depictions of hierarchical structures into two basic graphical forms: stacked and nested schemes. Whereas phylogenetic trees are stacked schemes with lines connecting the elements in their set, nested schemes depict elements as containers that are grouped and assembled according to their hierarchical relationships. One of the best known nested schemes is Ben Schneiderman’s “treemap” [17] which displays hierarchical data as a set of nested rectangles with areas that are proportional to a specified dimension of the data. There are several variations of treemap layouts: icicle, squarified, pivot by split size, Voronoi, radial icicle [16], and cascaded [18]. Each of these methods offers certain advantages over stacked schemes such as: (i) they use screen real estate more efficiently, (ii) they make it easier to recognize groups, patterns and outliers across large data sets, and (iii) they can convey multiple dimensions of data through size, colour, border quality and shape. While treemaps are useful for representing data categories that differ in their relative abundance, they can be difficult to interpret since the rectangular bins representing category abundance are typically of different shapes, and the level of bin nesting is often difficult to observe. Nevertheless, one treemap approach that has been successfully applied to phylogenetic visualization is MetaTreeMap [19], which uses the D3.js library [20].

**Fig 1:**
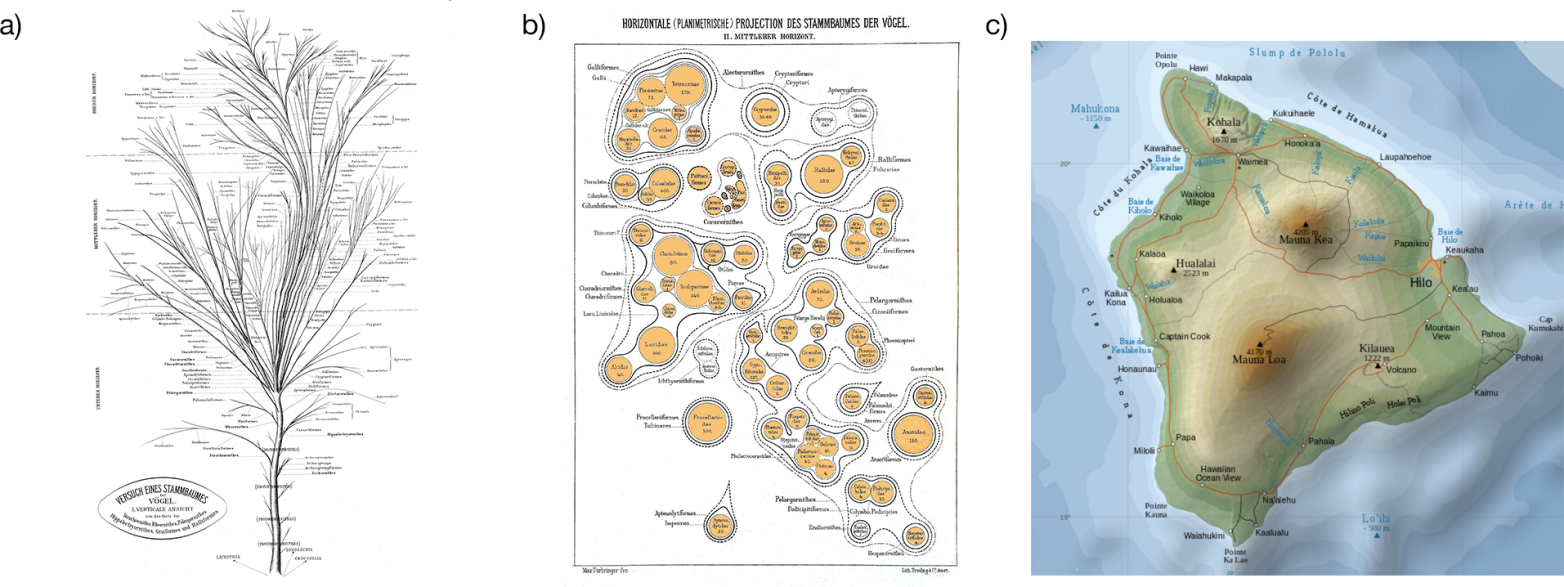
a) Max Fürbringer’s “Phylogenetic Tree of Birds”. b) A horizontal projection from the middle section of the tree. These figures, reproduced from https://en.wikipedia.org/wiki/Max_F%C3%BCrbringer, were originally published in 1888 and are in the public domain. c) A topographic map of Hawaii island, retrieved from https://en.wikipedia.org/wiki/Hawaii_(island)#/media/File:Hawaii_Island_topographic_map-en.svg. Reprinted with permission as per http://creativecommons.org/licenses/by-sa/3.0/.

We decided to explore a different approach to visualizing hierarchical information inspired by the work of Max Fürbringer, who in 1888 drew a phylogenetic tree of birds that included a series of “slices” from the middle of the tree [21] (Fig *1*A & *1*B). This graphic resembles a nested scheme called “circle packing” [22] however the bounding shapes are irregular, allowing for a much more efficient use of space. The visualization approach is also reminiscent of that used in topographic maps (Fig *1*C), where contour lines are used to indicate common levels of elevation, and consequently, higher levels of elevation are nested within lower levels. When applied in a phylogenetic context, phylogenetic depth (time to most recent common ancestry) is analogous to geospatial elevation, contour lines indicate the nested clustering of lineages and clades, and gaps between clusters indicate evolutionarily distinct clades.

The idea of using topographic maps to visualize hierarchical information has been explored by others. La Rosa et. al [23] demonstrate a method to create a topographic representation of bacteria clusters organized in a rectangular lattice that defines data neighborhood relationships. Unfortunately, the charts produced are non-intuitive and consequently difficult to decipher. A project by Cortese et. al [24] explores the use of topographic maps to visualize the connections between internet service providers and track the paths of information packets across a hierarchical network. Their overall approach is very appealing, but their topographic clustering is inverted with root and low level nodes getting drawn as high altitude peaks, while terminal and high level nodes are drawn at lower altitudes around the fringes.

## 2 Implementation

**Fig 2:**
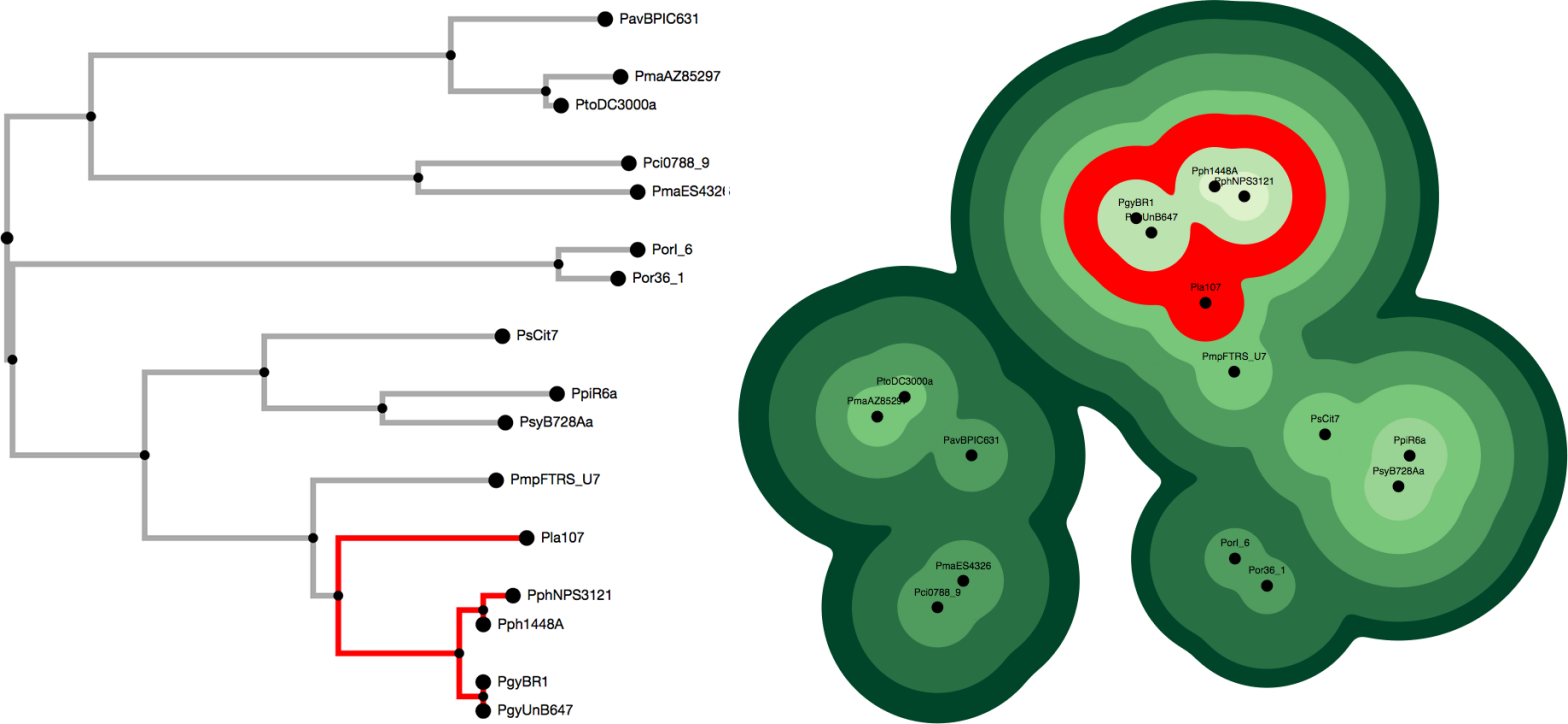
a) Phylogenetic tree with 16 OTUs. b) Topo-phylogeny chart based on the same structure. The equivalent branches are highlighted red in both figures.

We present a new tool for displaying phylogenetic relationships as a topographic map in which OTUs autonomously attract or repel one another based on their individual branch lengths and distance to a common ancestor to form “geographic regions” of related “peaks” (Fig 2). Topographic contour shapes indicate the level at which different OTUs are connected. The width of the contour around any given OTU indicates the branch lengths of each of its ancestors going back to the root. OTUs enclosed by the same contour shape share an ancestor. Valleys between the various plateaus and peaks indicate clades from a different lineage. We call this data visualization paradigm a “topo-phylogeny".

The program accepts data in Newick format, and can either be pasted into an input box or loaded from a file. All data processing is done within the browser on the client side. The Newick file gets parsed with Newick.js [25] and converted into a JSON object. This object is passed into d3.phylogram.js [26] to build a standard phylogenetic tree visible on the left side of the page.

On the right side of the page, the topo-phylogeny chart is created as an SVG image. Each node consists of a stack of circles, with the top circle containing the label and each circle below it sized according to the cumulative branch length at any given depth. Circle sizes are determined by area; radiuses are calculated as √(sum of branch lengths for that depth / π) with a minimum difference of 15 pixels and a maximum difference of 30 pixels from the next highest circle in the stack. The nodes determine their positions according to three basic principles: 1) Internal nodes are initially assigned "home" positions based on a radial phylogram algorithm. This ensures a good initial spacing so that clades don't begin in a crossed position. 2) All nodes are attracted to their siblings and relatives at different levels of strength. Internal nodes are also attracted to their home positions and children. 3) Terminal nodes repel one another based on a distance determined by the cumulative branch length of their common ancestor. Thus, nodes that are only related at the root repel each other at a distance based on the radius of their lowest (and biggest) circle, while sister nodes (i.e., they share the same parent) repel each other based on their highest (and smallest) circle. A force layout function triggers the attract and repel functions repeatedly until 100% of the nodes stop moving, at which point the force layout automatically stops.

**Fig 3:**
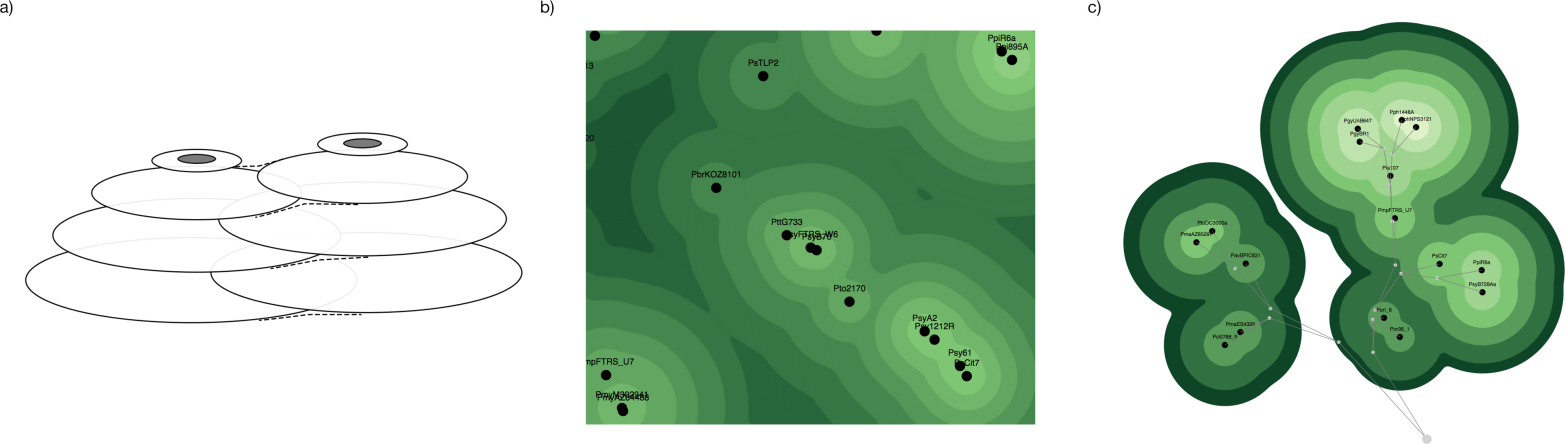
a) Nodes consist of stacks of interleaved SVG circle elements. b) Vector images can be scaled to any resolution. c) Links can be drawn above the nodes to help indicate the hierarchical structure.

SVG circles are grouped with other circles of their level within the same <g> tag. This makes it possible for several stacks of circles to interleave with one another and prevents higher level circles from getting covered by lower level circles (*Fig 3*A). A combination of blurring and sharpening SVG filters (commonly known as a "goo" effect) blends circles from the same level together, creating the perception of a single combined shape. This creates the topographic map effect with smooth shapes grouping OTUs of the same level. Because the shapes are drawn as vector images, it is possible to scale up the image to any resolution (Fig *3*B).

Internal nodes and “link lines” are drawn on top of the chart until the OTUs stop moving to help indicate the underlying relationships between the various items (Fig *3*C). This is a hybrid view, displaying the data using a combination of nested and stacked visual paradigms at the same time. These link lines can be toggled on and off with the “Show / Hide Links” button. They automatically shut off when the OTUs stop moving.

The position of the chart can be adjusted by dragging the canvas, and zoom settings can be adjusted using the mouse wheel. Individual OTUs can be dragged around the canvas if you would like to customize the layout. The “Save” button produces PNG and SVG images suitable for publication.

Topo-phylogeny is written with JavaScript, D3.js, jQuery and Materialize.css. Phylogenetic data should be in the Newick file format, either with or without bootstrap scores. Three example datasets of varying size are available for download at: http://bar.utoronto.ca. The source code can be downloaded from: https://figshare.com/s/15ce7763fcd97d778752.

## 3 Results

Topo-phylogeny charts with fewer than 20 OTUs take less than ten seconds to generate on a 2015 model 2.2 GHz Intel Core i7 MacBook Air with 8GB of RAM. Topo-phylogeny charts with more than a hundred OTUs can take several minutes before nodes settle into a stable position, however the major groupings are usually set quickly. Some topo-phylogeny charts may remain in constant motion if there is no ideal position for one or more of the OTUs. A ‘Pause’ button toggles the force layout function off and on if the user wishes to freeze the display. Hovering over the OTUs in either chart will highlight the equivalent OTU on both charts. This makes it possible to shift one’s gaze back and forth between both visualization methods without getting disoriented. Items of interest can be tagged with red or green markers by clicking or shift-clicking on them.

**Fig 4:**
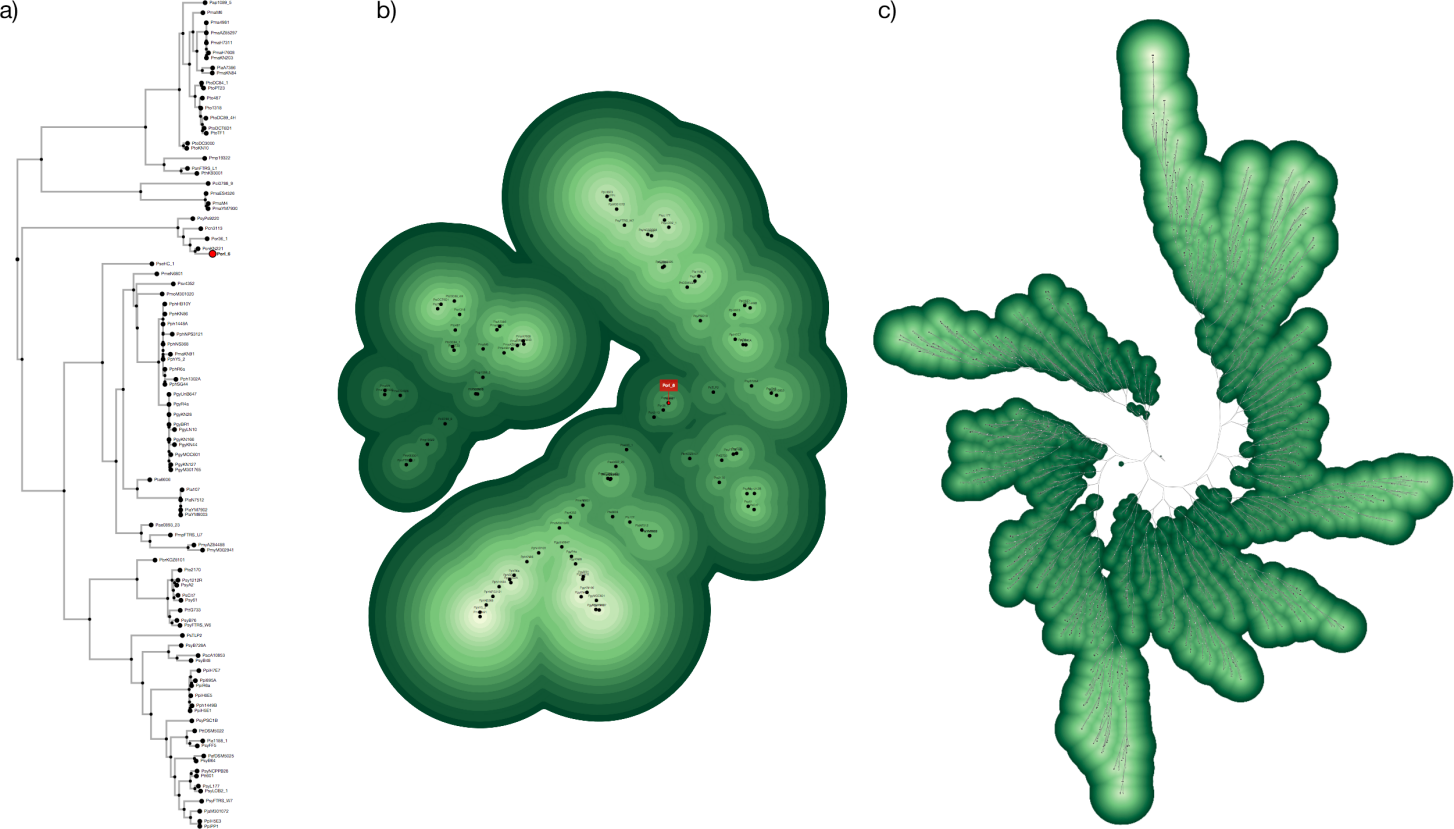
a) Phylogenetic tree with 96 OTUs. b) Topo-phylogeny chart based on the same structure. The same OTU is highlighted red in both figures. c) Topo-phylogeny chart based on Hug et al.’s Tree of Life (2016) with 3740 OTUs.

The technique scales well up to several hundred OTUs. Whereas large phylogenetic trees can be difficult to display and read, the underlying structure of a topo-phylogeny remains apparent (Fig *4*A & *4*B). Larger maps may have poor user interface response times because each node continuously compares its position against every other node, occupying system resources. Hug et al.’s Tree of Life [27] with 3740 OTUs and a maximum depth of 72 takes twenty minutes for the nodes to load, get drawn to the screen and find stable positions (Fig *4*C), but the program is virtually unresponsive during this time. The program includes an option to temporarily pause screen updates so the nodes can adjust their positions in the background without having to reposition all the DOM elements for each cycle. For large data sets, there is an option to adjust the “tightness” of the layout by changing a constant value (i.e., radius length) that is used to calculate the initial positions of each node. Future versions of the tool will focus on improving interactivity for very large data files.

## 4 Discussion

We present a new data visualization paradigm for exploring phylogenetic data. While topographic maps are a familiar method for displaying geospatial elevation data, their underlying visual paradigm can be equally well applied to other kinds of hierarchical data structures. Phylogenetic data are well suited to a topographic layout, and the use of topo-phylogeny charts can complement traditional phylogenetic approaches. Most notably, topo-phylogeny charts eliminate vertical scanning bias, where the proximity of OTUs at the tips of a tree is often confounded with their evolutionary relatedness–a mistake that is commonly made by individuals with limited training in phylogenetics. The free movement of nested clades in the two dimensional topo-phylogenetic space more accurately reflects the ability of nodes to freely rotate, thereby changing the relative position of OTUs, while retaining their nested evolutionary relationships.

Topo-phylogeny charts take advantage of preattentive visual processing [28] to help viewers grasp hierarchical relationships between items with a single glance. The end product looks like a topographic map in which each element’s position is determined by on its own attractions, repulsions and individual size. This visualization approach makes it easy to identify related and unrelated items because their group status preattentively “pops out” to the viewer. Items that are clustered together and surrounded by the same colour of contour shape are closely related; items that are in separate clusters and appear to have deep valleys between them are not. These relationships can be identified at a glance without having to trace individual branches back to the root.

Topo-phylogram is not intended as a replacement for the phylogenetic tree. Rather, we propose this data visualization paradigm as a complementary view. Viewing phylogenetic trees and topo-phylogeny charts side by side could improve viewers understanding of hierarchical structures because each method takes advantage of different visual processes.
